# Differential tissue accumulation of 2,3,7,8-Tetrachlorinated dibenzo-*p*-dioxin in *Arabidopsis thaliana* affects plant chronology, lipid metabolism and seed yield

**DOI:** 10.1186/s12870-015-0583-5

**Published:** 2015-08-11

**Authors:** Abdulsamie Hanano, Ibrahem Almousally, Mouhnad Shaban, Nour Moursel, AbdAlbaset Shahadeh, Eskander Alhajji

**Affiliations:** Atomic Energy Commission of Syria (AECS), B.P. Box 6091, Damascus, Syria; Department of Molecular Biology and Biotechnology, Atomic Energy Commission of Syria (AECS), P.O. Box 6091, Damascus, Syria; Department of Chemistry, Atomic Energy Commission of Syria (AECS), P.O. Box 6091, Damascus, Syria; Department of Protection and Safety, Atomic Energy Commission of Syria (AECS), P.O. Box 6091, Damascus, Syria

## Abstract

**Background:**

Dioxins are one of the most toxic groups of persistent organic pollutants. Their biotransmission through the food chain constitutes a potential risk for human health. Plants as principal actors in the food chain can play a determinant role in removing dioxins from the environment. Due to the lack of data on dioxin/plant research, this study sets out to determine few responsive reactions adopted by Arabidopsis plant towards 2,3,7,8-tetrachlorodibenzo-*p*-dioxin (TCDD), the most toxic congener of dioxins.

**Results:**

Using a high resolution gas chromatography/mass spectrometry, we demonstrated that Arabidopsis plant uptakes TCDD by the roots and accumulates it in the vegetative parts in a tissue-specific manner. TCDD mainly accumulated in rosette leaves and mature seeds and less in stem, flowers and immature siliques. Moreover, we observed that plants exposed to high doses of TCDD exhibited a delay in flowering and yielded fewer seeds of a reduced oil content with a low vitality. A particular focus on the plant fatty acid metabolism showed that TCDD caused a significant reduction in C18-unsaturated fatty acid level in plant tissues. Simultaneously, TCDD induced the expression of *9-LOX* and *13-LOX* genes and the formation of their corresponding hydroperoxides, 9- and 13-HPOD as well as 9- or 13-HPOT, derived from linoleic and linolenic acids, respectively.

**Conclusions:**

The current work highlights a side of toxicological effects resulting in the administration of 2,3,7,8-TCDD on the Arabidopsis plant. Similarly to animals, it seems that plants may accumulate TCDD in their lipids by involving few of the FA-metabolizing enzymes for sculpting a specific oxylipins “signature” typified to plant TCDD-tolerance. Together, our results uncover novel responses of Arabidopsis to dioxin, possibly emerging to overcome its toxicity.

**Electronic supplementary material:**

The online version of this article (doi:10.1186/s12870-015-0583-5) contains supplementary material, which is available to authorized users.

## Background

Polychlorinated dibenzo-*p*-dioxins (PCDDs) and polychlorinated dibenzofurans (PCDFs), collectively termed dioxins, are the most toxic group of Persistent Organic Pollutants (POPs). Composed of two aromatic rings linked via one (PCDFs) or two atom of oxygen (PCDDs) and one to eight related chlorine atoms, these halogenated chemicals are structurally very stable and extremely hydrophobic. Therefore, dioxins can persist in the environment and bioaccumulate at the top of food chain [1]. Humans may become exposed to dioxins mainly through food and less by inhalation or dermal contact. 2,3,7,8-Tetrachlorodibenzo-*p*-dioxin (TCDD), with a toxic equivalency factor (TEF) of 1.0, is the most toxic congener of dioxins. Consequently, TCDD was used as a good candidate for investigations of the physiological and toxicological effects of this class of chemicals [2–5].

In mammals, dioxins essentially accumulate in fats because of their high lipophilicity. For example, they reached maximal levels in liver adipose lipids and in milk lipid droplets [6]. In contrast low levels of these xenobiotics were measured in brain tissue [7]. The affinity of dioxins to lipids seems to be modulated by the biochemical nature of the particular lipids concerned. The accumulation of dioxins was observed to be highest in the lipid fraction composed of triglycerides than in those composed of phospholipids [8]. Also, it is well known that TCDD seriously affects lipid metabolism in exposed mammals. For example, exposure to TCDD increases membrane lipid oxidation and phospholipase (PLA2) activity, which in turn could increase the pool of free arachidonic acid (AA) [9, 10]. Moreover, exposure to TCDD may target AA metabolism downstream of PLA2 by inducing the enzymes which metabolize such fatty acids, the cytochrome P450, the cyclooxygenase and probably the lipoxygenase pathways [11, 12].

At the bottom of the food chain, plants are increasingly and persistently exposed to PCDD/Fs. Such xenobiotics cannot be used for nutrition or as a source of energy, but are nevertheless taken up and accumulated in plant tissues. PCDD/Fs-bioaccumulation in plant may has a serious impact on plant health, but also can contribute to bio-transmission of these xenobiotics to the top of food chain. These concerns led research efforts to focus on the biological capacity of plants to uptake contaminants from the soil via their roots and then translocate them into upper parts for storage, a mechanism called phytoextraction [13]. Due to their high hydrophobicity and low mobility, uptake of dioxins may not be readily accomplished by a passive diffusion in plants [14]. There are however, a very limited number of reports about the capacity of a few plants to uptake dioxins from the environment. For example, it has been documented that a variety of zucchini plant (*Cucurbita pepo* L.) accumulated various dioxin congeners and that their accumulation in roots depended on their hydrophobicity [15]. Uptake by plants of polychlorinated biphenyl (PCBs), few of them known also as dioxin-like compounds, has been more commonly reported. It has been found that some plant species, such as *Solidago canadensis*, *Vicia cracca*, *Chrysanthemum* sp*.*, and *Polygonumpersicaria* sp*.*, specifically transmitted PCBs into aerial parts and they are known as PCBs accumulators [16, 17].

In common with animals, plant lipids and their metabolites, mainly those derived from C18-unsaturated fatty acids are involved in many biological functions enabling plant to overcome biotic and abiotic stress including environmental pollutants [18, 19]. In contrast, the biological connections between dioxins and plant lipids remain largely unknown. We recently described that TCDD administration to Arabidopsis plant caused phytotoxicity effects including a decrease in seed germination, in fresh weight and in chlorophyll content, but it induced the formation of lateral roots. Additionally, the uptake of TCDD by Arabidopsis provoked an enhanced level of hydrogen peroxide H_2_O_2_ and a massive stimulation of anti-oxidative enzyme activities [20]. In the current study, three main issues were therefore addressed: i) Determination of the accumulation and translocation of TCDD in the tissues of Arabidopsis during a whole growth cycle. ii) Effect of TCDD on the chronology of principal growth stages of Arabidopsis and its consequent impact on plant yield. iii) As TCDD has a high affinity to lipids, modifications in fatty acids content and their peroxidation in Arabidopsis tissues and seeds after exposure to TCDD were demonstrated. Findings from this work will contribute to understand how plants respond to dioxins in the environment, a question which is of great importance.

## Results

### TCDD is up-taken and accumulated in Arabidopsis tissues

**Fig. 1:**
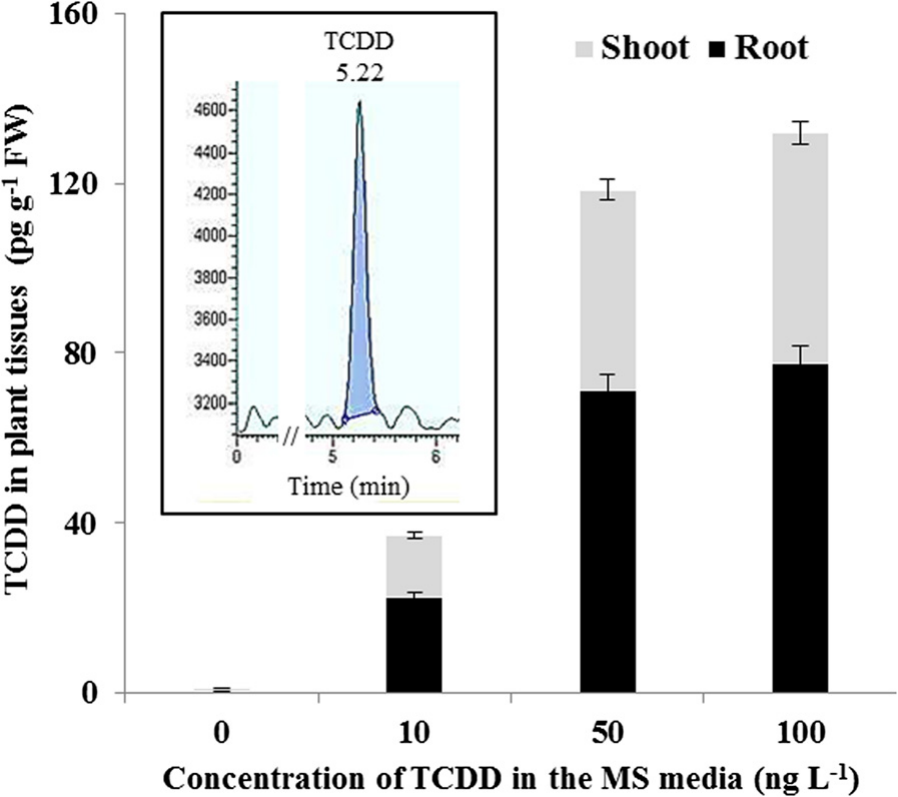
Detection and quantification of TCDD in Arabidopsis tissues by HR/GC-MS. Plants were grown for 30-days in glass tubes containing MS media supplemented with various concentrations of TCDD 0, 10, 50 and 100 ng L^−1^. In each treatment, shoots and roots of plant were taken and subjected separately to the extraction and analysis of TCDD. Concentration of TCDD in plant tissues was expressed as pg g^−1^ fresh weight. Inset, a representative HR/GC-MS diagram indicating the peak of TCDD (Retention time ≈ 5.22) in the plant extract. Three measurements were done for three individual plants. Data are mean values ± SD (*n* = 6)

HR/GC-MS diagrams presented in Additional file 1 show the presence of a single peak, corresponding to the TCDD (RT = 5.22 ± 0.4 min), in the organic extracts from the root of 30-days old Arabidopsis plants grown in the presence of various concentrations of TCDD 10, 50 and 100 ng L^−1^ (A, B and C, respectively). Similarly, the TCDD peak was also detected in the extracts from the shoot of the same plants (D, E and F). Compared to the standard curve of commercial TCDD (G), the content of TCDD in plant tissues paralleled its initial concentration in the media. Plants roots exposed to 10, 50 and 100 ng L^−1^ of TCDD contained 22.6 ± 1.5, 71.3 ± 2.2 and 77.6 ± 2.4 pg g^−1^ FW TCDD, respectively (Fig. 1), while TCDD-content in the shoots did not exceeded 14.4 ± 0.8, 47.1 ± 1.4 and 54.2 ± 2.0 pg g^−1^ FW. The recovery of TCDD from the spiked samples was approximately 97 % (data not shown). These results indicate that TCDD is taken up by the roots and subsequently translocated into the aerial parts of Arabidopsis.

### TCDD uptake depends on the growth stage of Arabidopsis

**Fig. 2:**
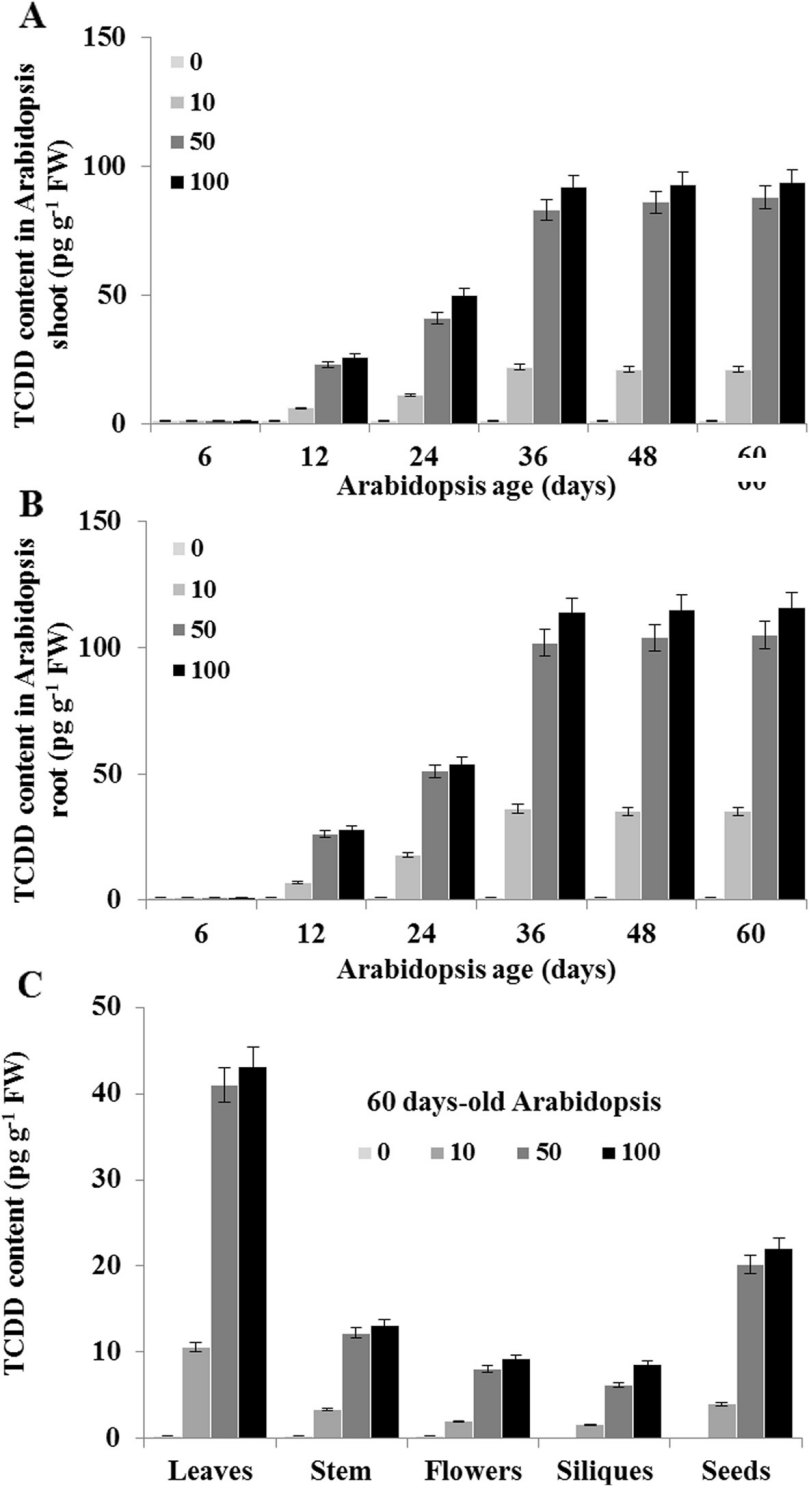
Differential accumulation of TCDD in various tissues of Arabidopsis as a function of the growth stage. Arabidopsis seeds were sown directly on TCDD-supplemented MS media. TCDD content was quantified in the shoot (**a**) and in the root (**b**) of Arabidopsis by HR/GC-MS at the indicated times. TCDD content was then quantified in the leaves, stem, flowers, siliques and seeds of 60-days old plants grown in the presence of 10, 50 and 100 ng L^−1^ TCDD (**c**). Three measurements were taken for three individual plants at indicate times. Values are mean ± SD (*n* = 6). FW: fresh weight

To determine a possible variation in the plant capacity to uptake the TCDD throughout the plant life cycle, we quantified the content of TCDD in Arabidopsis shoot and root at different stages of development, corresponding to 6, 12, 24, 48 and 60 days after TCDD administration. As shown in Fig. 2a, we started to detect the TCDD on day 12 in the shoot of Arabidopsis with its content reaching 6 ± 0.8, 23 ± 1.3 and 26 ± 1.5 pg g^−1^ FW. TCDD content had doubled on day 24 and tripled on day 36, then reached a plateau on day 48 post TCDD-exposure. Similarly, TCDD found to be detected in Arabidopsis root on day 12 and its accumulation tended to peak 36 days after treatment (Fig. 2b). Globally, the TCDD accumulation tended to be superposed in the shoots and roots of Arabidopsis throughout the plant life cycle. These data indicate that the TCDD uptake capacity of Arabidopsis varies throughout its life cycle and reaches its maximum on day 36.

### TCDD is accumulated mainly in leaves and seeds of Arabidopsis

We next evaluated the accumulation of TCDD in the upper parts of the Arabidopsis plant: i.e. leaves, stem, flowers, siliques and seeds. Leaves were the most active accumulators of TCDD, followed by seeds and siliques (Fig. 2c). Leaves accumulated TCDD at levels 5 to 6 times higher than stems and 10 to 15 times higher than flowers. Thus it seems that TCDD accumulates preferentially in leaves and seeds.

### TCDD shifts up the chronology of principal growth stages of Arabidopsis

**Fig. 3:**
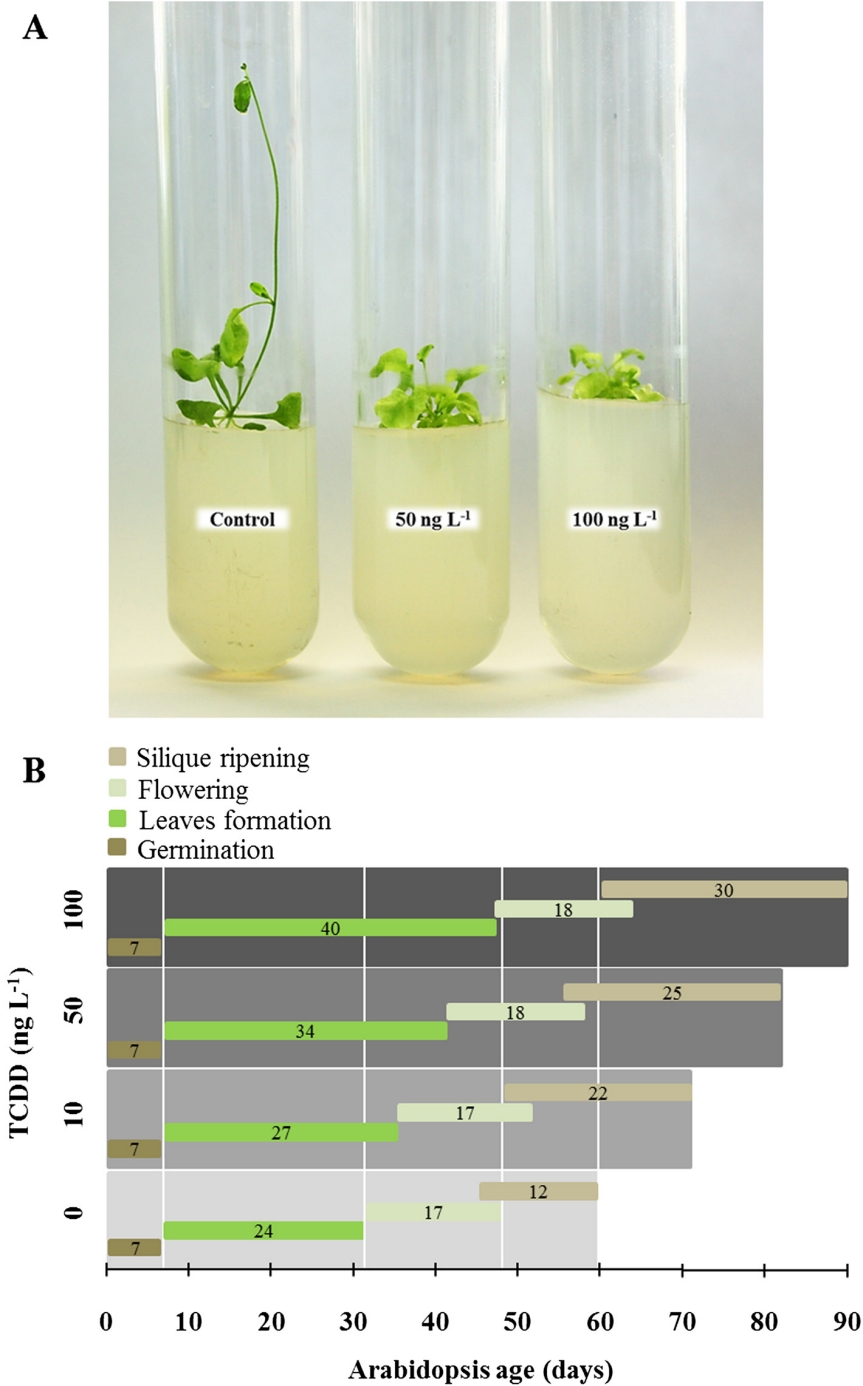
Chronological effect of TCDD on the principal growth stages of Arabidopsis plant. **a**. A representative image of plants growth in the presence or absence of TCDD under *in vitro* conditions. Seeds were directly sown into MS-tubes and left for germination as described before. Image was taken on day 30 after sowing. **b**. Presentation idea is inspired from Boyes et al., [44]. Plants were grown in the presence of TCDD with the indicated doses. Start-point and end-point for each stage of Arabidopsis development including germination, leaves formation, flowering and siliques ripening were determined according to Boyes et al. [44]. Measurements for each stage were taken for six individual plants. Data are mean values ± SD (*n* = 6)

We previously reported that TCDD had an inhibiting effect on the seed germination of Arabidopsis and affected the biomass and morphology of survival plants [20]. Here, we investigated the chronological effect of TCDD on the life cycle of the Arabidopsis plant including principal growth stages: i.e. germination, leaf formation, flowering and siliques ripening. Compared with controls, completion of the growth stages of TCDD-treated plants were delayed 13, 24 and 30 days when they were exposed to 10, 50 and 100 ng L^−1^ TCDD, respectively (Fig. 3). The stages of leaf formation and siliques ripening were especially affected with completion delayed 16 and 18 days at the highest dose of TCDD (100 ng L^−1^). Therefore the subsequent delay in flowering time (16 days) was marked. The duration of germination and flowering stages were not influenced by TCDD. Altogether, these data indicate that TCDD slows down the Arabidopsis growth cycle mainly by affecting leaf development and siliques maturation.

### TCDD affects both yield and oil content of Arabidopsis seeds

**Fig. 4:**
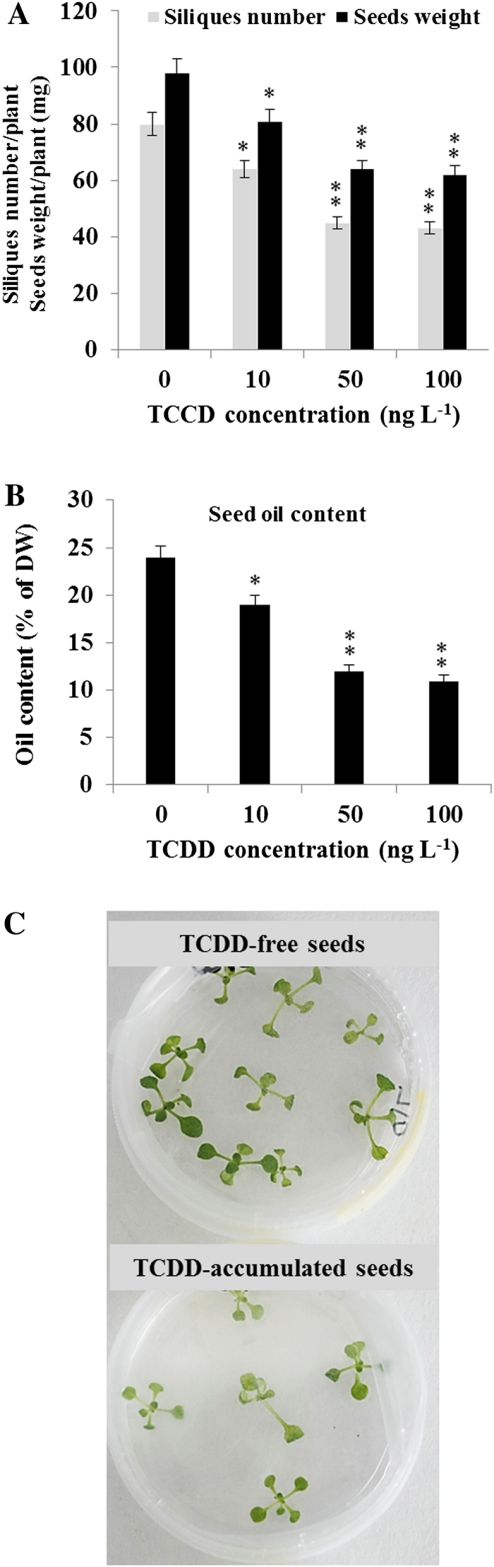
Effect of TCDD on seed yield of Arabidopsis. **a**. The final number of siliques per plant was determined after the completion of flower production. Yield is reported as the desiccated mass (mg) of seed produced per plant. **b**. Seed oil content is expressed as percentage of seed dry weight. **c**. Vitality of seeds produced from TCDD-treated plants compared with seeds of non-treated plants. Ten seeds were sown on MS-plate and left for germination as described before. Three measurements were taken for three individual plants. Data are mean values ± SD (*n* = 6). DW: dry weight. Three independent biological experiments were analyzed. Statistical significance of the data was evaluated by ANOVA analysis. Asterisks indicate significant differences between treatment and control: *P < 0.05 (significant); **P < 0.01 (very significant)

The effect of TCDD on the productivity of Arabidopsis was addressed. Seeds produced from TCDD-exposed plants were qualitatively and quantitatively examined. First, siliques number per plant decreased markedly from 80 siliques in non-exposed plants to 64, 45 and 43 siliques in TCDD-exposed plants at the indicated doses, as shown in Fig. 4a. Moreover, the mean weight of Arabidopsis seeds was seriously affected as a function of TCDD administration. The mean weight per plant was significantly reduced from 98 mg in control plants to 81, 64 and 61 mg in TCDD-exposed plants at indicated concentrations, respectively (Fig. 4a). Next, we examined the oil content in the harvested seeds as a relationship of TCDD-exposure. Figure 4b shows that seeds lost approximately 20, 50 and 54 % of their total oil as a result of TCDD treatment with the indicated concentrations. Furthermore, the vitality of seeds produced from the TCDD-treated plants have decreased by half compared with those produced from non-treated plants (Fig. 4c). These results indicate that exposure to TCDD yields plants with fewer seeds of a reduced oil content and a low vitality.

### TCDD affects lipid metabolism and induces a high level of lipid peroxidation in Arabidopsis

**Fig. 5:**
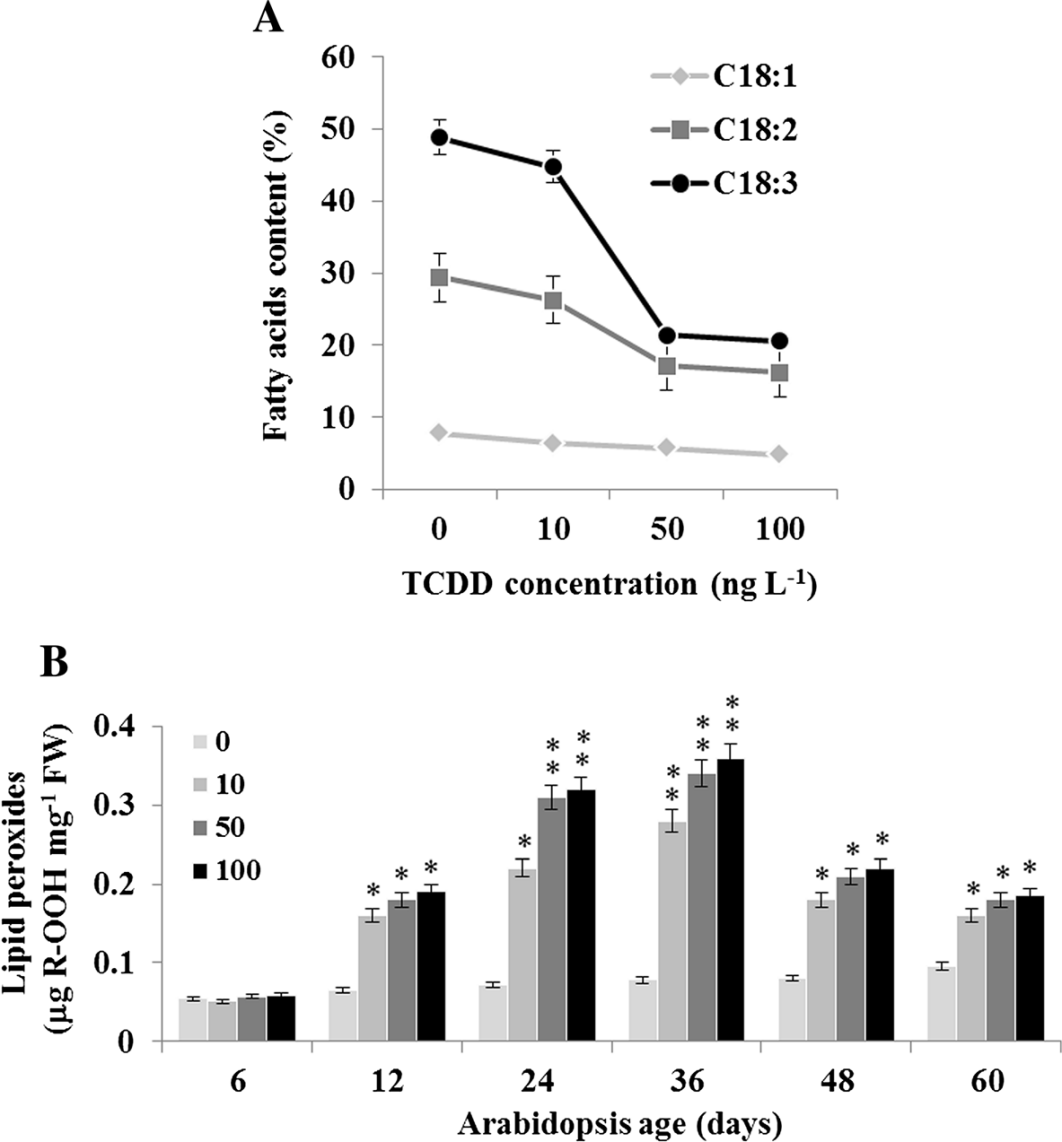
Lipid peroxidation as response to TCDD-exposure. **a**. Content of major C18-fatty acids (%) in Arabidopsis shoot on day 36 after exposure to TCDD at indicated doses. **b**. Total hydroperoxides produced by untreated and TCDD-treated Arabidopsis tissues was monitored using FOX-1 assay at various stage of development. Three independent plants were examined at each concentration of TCDD. Three measurements were taken per extract. Data are mean values ± SD (*n* = 6). FW: fresh weight. Statistical significance of the data was evaluated by ANOVA analysis. Asterisks indicate significant differences in lipid peroxides according to the plant age compared to germination stage (6 days): *P < 0.05 (significant); **P < 0.01 (very significant)

TCDD is known to affect lipids in mammals [10]. We investigated whether it has the same effect in plants by analyzing the content of the most abundant unsaturated fatty acids i.e. oleic (C18:1), linoleic (C18:2) and linolenic (C18:3) in post 36-day TCDD-exposed plants (Fig. 5a). TCDD exposure led to a decrease in the content of all these fatty acids with linolenic acid being the most affected. Compared with control, the content of C18:3 declined 2.3 fold in plant tissues after exposure to 50 ng L^−1^, while the contents of C18:2 and C18:1 were reduced 1.7 and 1.3 fold, respectively. These results suggest that exposure to TCDD provokes a net reduction of the C18-unsaturated FA content in Arabidopsis tissues. Moreover, it is well known that the exposure of plants to xenobiotic leads to lipid peroxidation [21]. To test this possibility, we measured the levels of total lipid peroxides over time in whole Arabidopsis plants after treatment with TCDD. The data in Fig. 5b shows that lipid peroxides increased progressively and significantly in TCDD-exposed plants aged between 24 and 36 days. Lipid peroxidation considerably declined in these plants on days 48 and 60 but remained slightly higher in the same tissues of control plants (Fig. 5b). These data suggest that exposure to TCDD induces lipid peroxidation in Arabidopsis tissues.

### TCDD-induced hydroperoxides are essentially derived from LOXs pathways

**Fig. 6:**
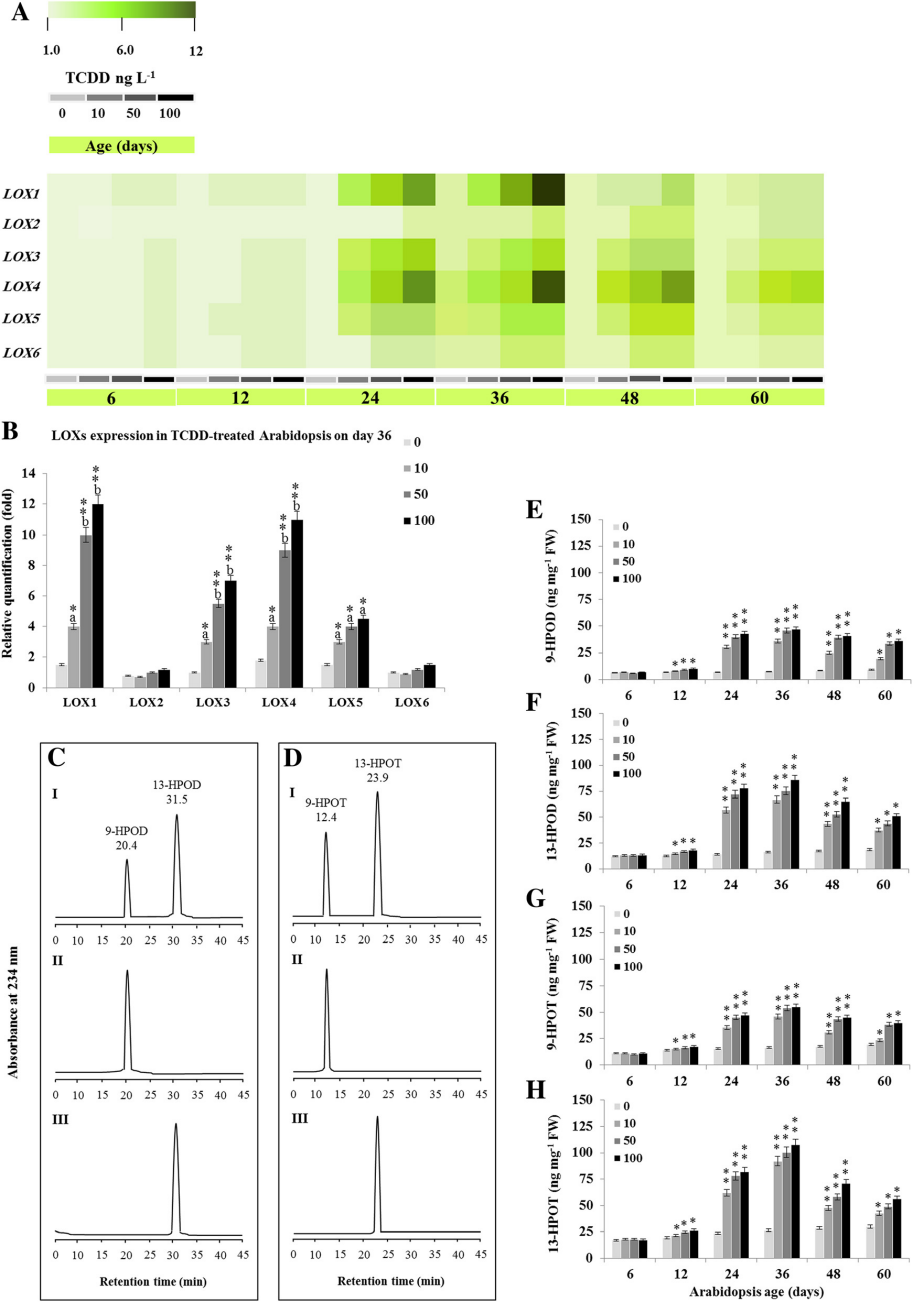
Accumulation of LOX-derived fatty acids hydroperoxides in TCDD-exposed Arabidopsis. **a**. Transcriptional fold changes of lipoxyganse encoding genes (*LOXs*) in whole plants after exposure to TCDD (0, 10, 50 and 100 ng L^−1^) for 6, 12, 24, 36, 48 and 60 days. For each gene, the transcript level was estimated by qRT-PCR as described in methods. **b**. Quantification of *LOX* genes expression in Arabidopsis tissues after treatment with indicated concentrations of TCDD for 36 days. Three measurements were taken in three cDNAs prepared from three individual plants for each TCDD-treatment. Different lowercase letters indicate significant differences in the expression for each *LOX* gene according to various concentrations of TCDD and control: ^a^ *P* < 0.05 (significant); ^b^ *P* < 0.01 (very significant). Asterisks indicate significant differences between the expression of *LOXs* genes according to each treatment: *P < 0.05 (significant); **P < 0.01 (very significant). **c** and **d**. UV-HPLC**-**separation of 9- or 13-HPOD and 9- or 13-HPOT extracted from TCDD-exposed Arabidopsis tissues at different stages of development. **e**, **f**, **g** and **h**. The four major hydroperoxide fatty acids (9-HPOD, 13-HPOD, 9-HPOT, and 13-HPOT) of Arabidopsis were extracted and quantified as described in methods. For each hydroperoxide, three measurements were taken in three individual plants. Data are mean values ± SD (*n* = 6). FW: fresh weight. Statistical significance of the data was evaluated by ANOVA analysis and Duncan’s multiple range test. Asterisks indicate significant differences in lipid peroxides according to the plant age compared to germination stage (6 days): *P < 0.05 (significant); **P < 0.01 (very significant)

Unsaturated fatty acid hydroperoxides can be formed either chemically [22] or enzymatically under the action of α-dioxygenases (α-DOX) [23] and lipoxygenases (LOX) [24]. Arabidopsis contains six genes encode *LOXs* [25] and at least two genes encode *α-DOXs* [26]. Transcriptional analysis of *LOXs* genes showed an up-regulation of *LOX1*, *LOX4*, *LOX3* and *LOX5* in whole plants aged 36 days after exposure to TCDD, whereas the expression levels of *LOX2* and *LOX6* were not affected (Fig. 6a). The expression of *LOX1*, *LOX4*, *LOX3* and *LOX5* was significantly increased in treated plants for 36 days compared to control and reached about 12, 11, 7 and 4.5 fold, respectively (Fig. 6b). Accordingly, the hydroperoxides deriving from C18:2 and C18:3, under 9-LOX and 13-LOX catalysis, accumulated and reached their maximum on day 36 then declined on days 48 and 60. The accumulation of hydroperoxide derivatives from C18:2 were higher than those of C18:3, the highest being 9-HPOD (6.2 fold) followed by 13-HPOD (5.1 fold) then 13-HPOT (4.0 fold) and 9-HPOT (3.3 fold) as shown in Fig. 6c, d, e, f, g and h. These results taken together suggest that the exposure of Arabidopsis to a high dose of TCDD leads to the accumulation of fatty acid hydroperoxides probably resulting from the up-regulation of *LOX* genes expression.

**Fig. 7:**
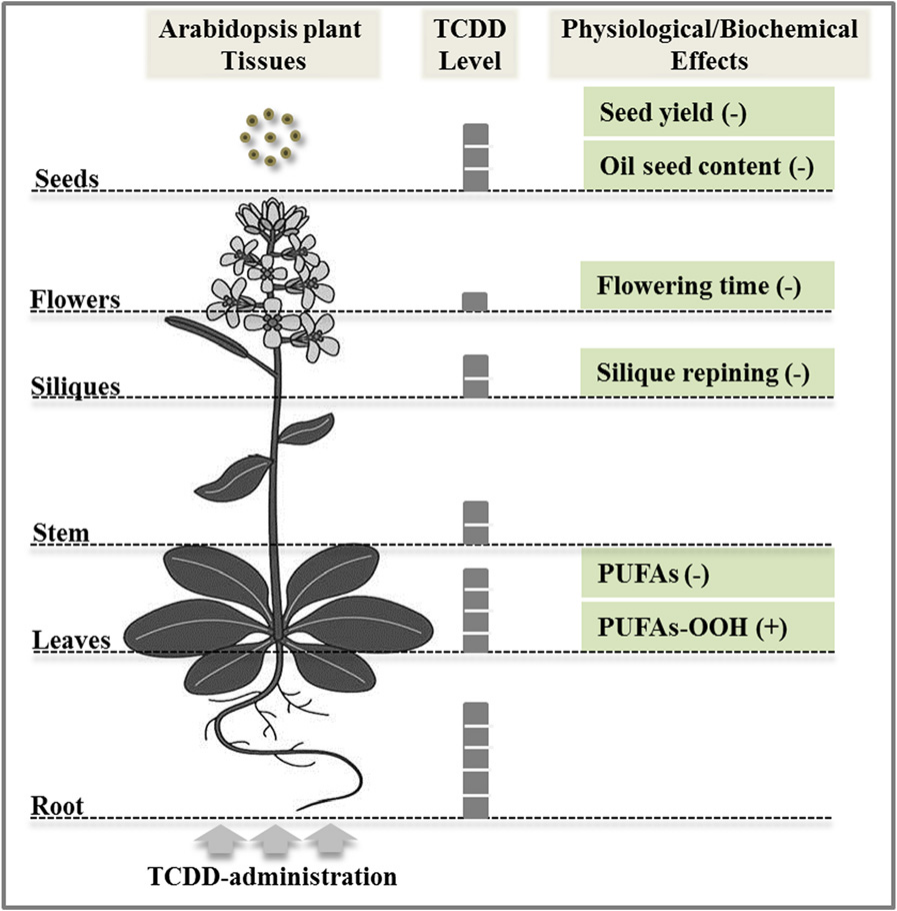
Representative schema for TCDD accumulation and its subsequent physiological and biochemical effects on Arabidopsis. TCDD is up-taken by the root and accumulated into the upper parts especially in the leaves and the mature seeds. The salient biological features affected by TCDD-exposed plant are: flowering time, siliques repining and seeds yield. Polyunsaturated fatty acids (PUFAs) and their metabolism are seriously alerted by TCDD. Thus, the role of lipid metabolism as a response to TCDD-exposure in plant merits a particular focus

## Discussion

We have recently reported that the immediate uptake of 2,3,7,8-TCDD by Arabidopsis yielded various phytotoxicological effects [20]. Herein, we have shown that the accumulation and translocation of TCDD in Arabidopsis plant depended on the growth stage. Moreover, TCDD-exposed Arabidopsis plants were affected in their lipid metabolism, flowered late and produced less seeds than non-exposed plants. These results are summarized in Fig. 7.

Using HR/GC-MS analysis, we showed that TCDD-accumulation in Arabidopsis plants was ordered in a tissues-specific manner, mainly in the root and less in the shoot. These estimations confirm our previous data [20] and come in line with previous reports on the natural ability of various plant species to take up dioxins/furans from their environment [27–30]. From the upper parts, we observed that the highest levels of TCDD were found in leaves and mature seeds of Arabidopsis. The accumulation of TCDD in seeds can be explained by the high affinity of TCDD toward lipids that are abundantly present in Brassicaceae seeds [31]. Indeed, it is well known that dioxins essentially bioaccumulate in animal fats because of their high lipophilicity [6]. Accordingly, dioxin and dioxin-like compounds were often detected in rape and olive oils [32, 33]. Moreover, the sorption of hydrophobic organic compounds (HOC) by lipid bodies of rapeseeds as a HOC-removal strategy is well documented [34, 35]. Similarly, the high concentration of TCDD in leaves might result from the high affinity of TCDD for the lipid-membrane of chloroplasts, mitochondria and peroxisomes. In this context, it is known that the major xenobiotic-oxidations catalyzed by enzymatic systems take place in the endoplasmic reticulum and in the membrane of chloroplasts and peroxisomes [36]. Accordingly, data published from proteomic analyses carried out on whole tissue/organ preparations of Arabidopsis revealed a total of 265 environmental stress responding proteins. Most of them were located in chloroplast, mitochondria and peroxisome [37]. Thus, accumulation of dioxins in vegetative tissues and in seeds may reflect their biological fate in plants and suggest a possible tissue-specific mechanism for accumulation and subsequent detoxification of TCDD in plants.

A point of a particular importance is the mechanism responsible of translocation of TCDD in plant. Although the molecular and biochemical mechanisms involved in reception, translocation, genes activation and enzymes metabolism of TCDD are well documented in mammals, no data however are available to date about these mechanisms in plants. As many of xenobiotics including dioxins are highly lipophilic, they can accumulate to toxic levels in the plant tissues, unless effective means of detoxification are present. In the case of TCDD, one of the most active detoxification systems in plants can probably disposed of this xenobiotic by two sequential processes: a chemical transformation and a subsequent compartmentation [38]. The chemical transformation of lipophilic xenobiotics in plants is of two types: phase I, the activation reactions and phase II, the conjugation reactions. The primary function of the phase I is to create reactive sites in the xenobiotic by the addition of functional groups (e.g. hydroxyl or carboxyl) which make it more hydrophilic and prepare it therefore for phase II reactions, the conjugation to glutathione [38]. After having achieved these two protective phases, such xenobiotics are transported and accumulated in apoplastic cell walls or central vacuoles in plant cells. Biochemical, molecular, and genetic evidences have been reported on the functions of a handful of ATP-binding cassette and multidrug and toxic compound extrusion family transporters engaged in transport of organic xenobiotics [39]. From them, P-glycoprotein, identified in plants [40] as a vacuolar glutathione-conjugate transporter, has some attractions to be involved in TCDD transportation. Another possible and potential transporter of TCDD can be a protein, called MRP (Multidrug Resistance-associated Protein), which is recognized as a glutathione-conjugate transporter [41, 42]. However, the nature and the mechanism of TCDD transportation is still yet uncharacterized.

A chronological effect on the principle growth stages has been also observed. Regardless of the level of exposure, our results showed that the TCDD-content was maximal on day 36 post-administration. At this developmental stage, Arabidopsis possesses a complete rosette growth and an initial emergence of inflorescence. The high level of TCDD found in plant tissues at this stage of growth could be explained by the optimal development and extension of the lateral roots of Arabidopsis which seemed to be induced by TCDD [43]. Moreover, the biomass of vegetative tissues reached its maximum in this stage [20, 44]. Absorption of TCDD delayed flowering time (up to 16 days at highest dose of TCDD) through presently undetermined mechanisms. One possible mode of action of TCDD might be linked to its up-regulation of various MYB transcription factor genes in the root of Arabidopsis [20]. Flowering time is in part regulated through the transcriptional regulation of Flowering Locus T (FLT). Interestingly, it was recently reported that a MYB transcription factor Early Flowering MYB protein (EFM) plays an important role in directly repressing FLT expression in the root and leaf vasculature under normal conditions [45]. Following this hypothesis, the activating of MYB factors by TCDD would repress FLT and thus delays flowering. Alternatively, the strong decline of C18:3 might indicate a role of PUFAs in response to TCDD. In particular, TCDD might act through oxidized lipids. We observed here that the amount of lipid peroxides tripled 36 days after exposure to TCDD, this effect seems similar to the previous increase level of lipid peroxides in suspensions of tobacco cells when submitted to dioxins exposure [28]. Lipid oxidation is derived from chemical and enzymatic processes [24]. However the parallel rise in transcripts levels of *9-LOX* and *13-LOX* genes with the accumulations of 9- and 13-OOH deriving from linolei (n) ic acids suggests that the Arabidopsis response to TCDD occurred predominantly via LOX pathway. The involvement of the LOX pathway in responses of plants to tress-induced senescence [46] and heavy metals tolerance [47] has been reported. The LOX pathway is also involved during the regulation of lateral root development and flowering [48]. In particular, transition from vegetative growth to flowering in Arabidopsis was associated with the accumulation of 13-HPOT, resulting from the oxidation of linolenic acid by LOX [49]. It is tempting to hypothesize that the delay of accumulation of 13-HOO-FA in TCDD-treated plants might postpone flowering time. In addition oxylipins deriving from the reduction of 13-OOH-FA might play a role in the control of flowering transition. Indeed, overexpression of the caleosin/peroxygenase RD20 that catalyzes the formation of OH-FA led to early flowering whereas plants deprived of this caleosin flowered later than the control [50].

## Conclusions

In conclusion, as Fig. 7 summarized, the current work highlights a side of toxicological effects related to the administration of 2,3,7,8-TCDD on the Arabidopsis plant. In a tissue-specific manner, the highest TCDD levels were detected in rosette leaves and mature seeds and affected lipid metabolism. Similarly to animals, plants may accumulate TCDD in their lipids by involving few of the FA-metabolizing enzymes for sculpting a new oxylipins “signature” typified to plant TCDD-tolerance. Together, our results will contribute to a better understanding of the mechanisms adopted by plants in response to dioxin contamination, and therefore, these potential strategies protect the plants as well as their environment.

## Methods

### Arabidopsis culture conditions and TCDD treatment

*Arabidopsis thaliana* ecotype Columbia 0 (Col0) seeds were sterilized with 70 % alcohol and spread on solid Murashige and Skoog (Duchefa Biochimie, Netherlands) medium supplemented with 1.5 % sucrose. 2,3,7,8-tetrachlorodibenzo-p-dioxin (2,3,7,8-TCDD dissolved in toluene at 10 μg mL^−1^, purity 99 %) was purchased from Supelco Inc., USA. Arabidopsis seeds were sown directly into glass tubes (30 cm length × 2.5 cm diameter) containing 25 mL of MS media already prepared, autoclaved and supplemented with 2,3,7,8-TCDD at various concentrations 0, 10, 50 and 100 ng L^−1^. Toluene was added into the control plate to account for the effects of this solvent. Culture conditions were adjusted as we previously described [20]. Responses to TCDD were analyzed along of plant life cycle. To determine the chronological effect of TCDD, the timing and therefore the period of the main four stages of plant development including germination, leaves formation, flowering and siliques ripening were measured in the presence or absence of TCDD. The Start-point and end-point for each stage were determined according to Boyes et al., [44]. Representative plant tissues were separately frozen in liquid nitrogen and kept at – 80 °C for TCDD extraction procedures.

### Extraction and cleanup of TCDD from plant tissues

The extraction and cleanup of TCDD were carried out as described before [20]. In brief, approximately two grams of plant tissues were ground in liquid nitrogen with a ceramic mortar and pestle. Powders were mixed with 2 mL of 37 % HCl and 5 mL of 2-propanol, homogenized and then extracted with 3 mL of hexane by shaking vigorously overnight. After a brief centrifugation, the organic phase was taken for a second extraction for an hour. The combined extracts were evaporated to dryness and re-dissolved in 1 mL of hexane, acidified with 125 μL HCl (2 M) and then extracted twice with 1 mL of hexane. The extract were cleaned up with the small column (0.5 g anhydrous Na_2_SO_4_ on top, 1.0 g of florisil at the bottom). This column was activated with 3 mL of dichloromethane (DCM)/hexane/methanol (50:45:5) and then with 5 mL DCM/hexane/methanol of for elution. The eluates were evaporated to dryness and dissolved in 100 μL hexane for GC/MS analysis. A spiked sample with a known concentration of TCDD (50 ng mL^−1^) was done to validate our extraction and purification procedure.

### TCDD analysis by HR-GC/MS

2,3,7,8-TCDD content was quantified in the cleaned extracts of plant tissues of Arabidopsis by GC/MS using an Agilent Technologies 7890 GC System (USA) coupled to an AMD 402 high resolution mass spectrometer (Germany). Details of the MS analysis and quality control are described in EPA methods 1613B and 1668A. One-μL aliquot of the sample was injected into an Agilent DB-5 MS fused silica capillary column (60 m × 250 μm ID, film thickness 0.25 μm) with helium as carrier gas at a constant flow rate of 1.6 mL min^−1^. The oven temperature program was as described by Shen et al., [51] as following: start at 150 °C, held for 1 min, increased to 200 °C at 12 °C min^−1^, increased to 235 °C at 3 °C min^−1^ and held for 8 min, and finally increased to 290 °C at 8 °C min^−1^ and held for 20 min. Quantification was performed using an isotope dilution method.

### Total lipid extraction and fatty acid quantification

Mature seeds were harvested from TCDD-treated or control plants and analyses were performed on 2 mg of dried seeds. For plant tissues lipid analysis, 36-day old plant shoot was taken from control and TCDD-treated plants (100 mg). Total fatty acid was extracted with chloroform/methanol (1:2) (v/v) and were then methylated using 1 % (v/v) sulfuric acid in methanol at 100 °C for 2 h, as described previously [52]. The resulting fatty acid methyl esters (FAMEs) were extracted in hexane and analyzed by a GC-MS (Agilent 6850) as described previously by Murayama et al., [53]. FAs were identified and their relative amounts were calculated from their respective chromatographic peak areas compared with a FAME mixture used as a fatty acid standard. Seed lipids content was expressed as percentage of dry weight and the content in FAs was related to the fresh weight of plant tissues and later transformed into percentages of the total fatty acids obtained as described previously [43].

### Lipid peroxides quantification

FOX-1 assay was applied to monitoring the total hydroperoxides produced by untreated and TCDD-treated Arabidopsis tissues as described by Jiang et al., [54]. Total hydroperoxides was analyzed spectrophotometrically assay by measuring the oxidation of xylenol orange (FOX-1) at 560 nm.

### Fatty acid hydroperoxides characterization

Fatty acids hydroperoxides were extracted from Arabidopsis tissues and analyzed according to Göbel et al., [55] with brief modifications. Two grams of plant tissues were immediately ground in liquid nitrogen. After adding 10 mL of extraction solvent (*n*-hexane:2-propanol, 3/2 (v/v) with 0.025 % (w/v) butylated hydroxytoluene), mixture was immediately ultra-homogenized for 30 s on ice. A spiked sample with 10 μM of each hydroperoxide was used as a control. The extract was then shaken for 10 min and centrifuged at 3,000 × *g* at 4 °C for 10 min. The upper phase was collected, and a 6.7 % (w/v) solution of potassium sulfate was added up to a volume of 16.2 mL. After vigorous shaking and a brief centrifugation at 4 °C for 10 min the upper layer was subsequently taken and dried under streaming nitrogen. Hydroperoxides were taken within 25 μL of acetonitrile/water/acetic acid (50/50/0.1) (v/v/v) and their quantification were carried out on a Jasco LC-2000 plus series HPLC system (Jasco, USA) using a UV-detector (RF-10Axl, Shimadzu) (234 nm) and a C18 column (Eclipse XDB-C18 150 × 4.6 mm, 5 μm; Agilent, USA). The analysis was performed using a mobile phase of acetonitrile/water/acetic acid (50/50/0.1, v/v/v) at a flow rate of 0.6 mL min^−1^. FA-hydroperoxides were quantified using their respective standards.

### RNA extraction, reverse transcription and quantitative RT-PCR

Two grams of plant material were used to total RNA extraction using an RNeasy kit according to the manufacturer’s instructions (Qiagen, Germany). Quality of extracted RNAs was controlled on agarose gel and their concentration was measured by Nanodrop (Nano Vue, GE Healthcare). Reverse transcription reaction (RT) was carried out according to Hanano et al., [56]. Real-time PCR was performed in 48-well plates using a StepOne cycler from Applied Biosystems, USA as described by Czechowski et al. [57]. Briefly, 25 μL reaction mixtures contained 0.5 μΜ of each specific oligonucleotide primer for the target (*LOXs*) and the reference genes (*SAND* and *TIP41*) (Table 1), 12.5 μL of SYBR Green PCR mix (Bio-Rad, USA) and 100 ng cDNA. QPCR conditions were as described before [20]. The relative expression of target genes was normalized using two reference genes *SAND* and *TIP41* [58]. Each point was replicated in triplicate and the average of *C*_T_ was taken. Subsequently, the relative quantification RQ of *LOXs* gene was calculated directly by the software of the qPCR system. The sequences of amplified regions were confirmed by sequencing on an ABI 310 Genetic Analyzer (Applied Biosystems) using Big Dye Terminator kit (Applied Biosystems).

**Table 1:** List of primers used in this study

Gene	AGI	Amplicon (bp)	Forward primer (5'-3')	Reverse primer (5'-3')
LOX1	At1g55020	168	CACATGAAACACCAGCGACG	GTGTCCCTCCAAGTACAGGC
LOX2	At3g45140	190	CGATGTTGGTGACCCTGACA	TGAAGTGCCCTTGGCTGTAG
LOX3	At1g17420	143	ACGACCTTGGAAATCCCGAC	TGGCTTCTCTACTCGGCTCT
LOX4	At1g72520	152	GGCGGGTGGAGAAACCATTA	AGCGAAGTCCTCAGCCAAAA
LOX5	At3g22400	111	GGCTCTCCCAAAAGACCTCC	TCTAAACCGTCGACCGCAAA
LOX6	At1g67560	198	TTCGGACAGTACCCGTTTGG	GTCAGGGGAATGCGTTGAGA
DIOX	At3g01420	113	AGACATTGTTCCCCACGACC	TGAACTCGTTGTACCGTGGG
SAND	AT2G28390	76	GGATTTTCAGCTACTCTTCAAGCTA	CTGCCTTGACTAAGTTGACACG
TIP41	AT4G34270	96	GAACTGGCTGACAATGGAGTG	ATCAACTCTCAGCCAAAATCG

### Statistical analysis

All data presented were expressed as means ± standard deviation (SD). Statistical analysis was performed using STATISTICA software, version10 (StatSoft Inc.). Comparisons between control and treatments were evaluated by ANOVA analysis and multiple comparisons, Duncan’s multiple range test. Difference from control was considered significant as *P* < 0.05 or very significant as *P* < 0.01.

## Additional file

Additional file 1:
**Detection of TCDD in Arabidopsis tissues by HR/GC-MS**. Diagrams indicate the presence of TCDD (Retention time ≈ 5.22) in the root (A, B and C) and in the shoot (D, E, and F) of 30-days old Arabidopsis exposed to various concentrations of TCDD 10, 50 and 100 ng L^−1^, respectively. G. TCDD standard. Three measurements were taken for three individual plants. Data are mean values ± SD (*n* = 6). (PDF 204 kb)

## Abbreviations

9-HPOD: 9 (S)-hydroperoxyoctadecadienoic acid
13-HPOD: 13 (S)-hydroperoxyoctadecadienoic acid
9-HPOT: 9 (S)-hydroperoxyoctadecatrienoic acid
13-HPOT: 13 (S)-hydroperoxyoctadecatrienoic acid
TCDD: 2,3,7,8-polychlorinateddibenzo-p-dioxins
PCDDs: Polychlorinated dibenzo-*p*-dioxins
PCDFs: Polychlorinated dibenzofurans
PUFAs: Polyunsaturated fatty acids
TEF: Toxic equivalency factor

## References

[1] Pollitt F (1999). Polychlorinated dibenzodioxins and polychlorinated dibenzofurans. Regul Toxicol Pharmacol.

[2] Poland A, Knutson JC (1982). 2,3,7,8-tetrachlorodibenzo-p-dioxin and related halogenated aromatic hydrocarbons: examination of the mechanism of toxicity. Annu Rev Pharmacol Toxicol.

[3] Huuskonen H, Unkila M, Pohjanvirta R, Tuomisto J (1994). Developmental toxicity of 2,3,7,8-tetrachlorodibenzo-p-dioxin (TCDD) in the most TCDD-resistant and -susceptible rat strains. Toxicol Appl Pharmacol.

[4] Baker TK, Kwiatkowski AP, Madhukar BV, Klaunig JE (1995). Inhibition of gap junctional intercellular communication by 2,3,7,8-tetrachlorodibenzo-p-dioxin (TCDD) in rat hepatocytes. Carcinogenesis.

[5] Lindén J, Lensu S, Tuomisto J, Pohjanvirta R (2010). Dioxins, the aryl hydrocarbon receptor and the central regulation of energy balance. Front Neuroendocrinol.

[6] Ulaszewska MM, Zuccato E, Davoli E (2011). PCDD/Fs and dioxin-like PCBs in human milk and estimation of infants' daily intake: a review. Chemosphere.

[7] Ishida T, Matsumoto Y, Takeda T, Koga T, Ishii Y, Yamada H (2010). Distribution of 14C-2,3,7,8-tetrachlorodibenzo-p-dioxin to the brain and peripheral tissues of fetal rats and its comparison with adults. J Toxicol Sci.

[8] Lovati MR, Galbussera M, Franceschini G, Weber G, Resi L, Tanganelli P (1984). Increased plasma and aortic triglycerides in rabbits after acute administration of 2,3,7,8-tetrachlorodibenzo-p-dioxin. Toxicol Appl Pharmacol.

[9] Al-Bayati ZA, Stohs SJ (1991). The possible role of phospholipase A2 in hepatic microsomal lipid peroxidation induced by 2,3,7,8-tetrachlorodibenzo-p-dioxin in rats. Arch Environ Contam Toxicol.

[10] Lawrence BP, Kerkvliet NI (1998). Role of altered arachidonic acid metabolism in 2,3,7, 8-tetrachlorodibenzo-p-dioxin-induced immune suppression in C57Bl/6 mice. Toxicol Sci.

[11] Rifkind AB, Gannon M, Gross SS (1990). Arachidonic acid metabolism by dioxin-induced cytochrome P-450: a new hypothesis on the role of P-450 in dioxin toxicity. Biochem Biophys Res Commun.

[12] Gilday D, Bellward GD, Sanderson JT, Janz DM, Rifkind AB (1998). 2,3,7,8-tetrachlorodibenzo-p-dioxin (TCDD) induces hepatic cytochrome P450-dependent arachidonic acid epoxygenation in diverse avian orders: regioisomer selectivity and immunochemical comparison of the TCDD-induced P450s to CYP1A4 and 1A5. Toxicol Appl Pharmacol.

[13] Cherian S, Oliveira MM (2005). Transgenic plants in phytoremediation: recent advances and new possibilities. Environ Sci Technol.

[14] Collins C, Fryer M, Grosso A (2006). Plant uptake of non ionic organic chemicals. Environ Sci Technol.

[15] Inui H, Wakai T, Gion K, Yamazaki K, Kim YS, Eun H (2011). Congener specificity in the accumulation of dioxins and dioxin-like compounds in zucchini plants grown hydroponically. Biosci Biotechnol Biochem.

[16] Zeeb BA, Amphlett JS, Rutter A, Reimer KJ (2006). Potential for phytoremediation of polychlorinated biphenyl-(PCB-) contaminated soil. Int J Phytoremediation.

[17] Ficko SA, Rutter A, Zeeb BA (2010). Potential for phytoextraction of PCBs from contaminated soils using weeds. Sci Total Environ.

[18] Howe GA, Schilmiller AL (2002). Oxylipin metabolism in response to stress. Curr Opin Plant Biol.

[19] Eckardt NA (2008). Oxylipin signaling in plant stress responses. Plant Cell.

[20] Hanano A, Almousally I, Shaban M (2014). Phytotoxicity effects and biological responses of *Arabidopsis thaliana* to 2,3,7,8-tetrachlorinated dibenzo-p-dioxin exposure. Chemosphere.

[21] Baryla A, Laborde C, Montillet JL, Triantaphylidès C, Chagvardieff P (2000). Evaluation of lipid peroxidation as a toxicity bioassay for plants exposed to copper. Environ Pollut.

[22] Mosblech A, Feussner I, Heilmann I (2009). Oxylipins: structurally diverse metabolites from fatty acid oxidation. Plant Physiol Biochem.

[23] Hamberg M, Sanz A, Castresana C (1999). Alpha-oxidation of fatty acids in higher plants: identification of a pathogen-inducible oxygenase (piox) as an alpha-dioxygenase and biosynthesis of 2-hydroperoxylinolenic acid. J Biol Chem.

[24] Feussner I, Wasternack C (2002). The lipoxygenase pathway. Annu Rev Plant Biol.

[25] Bannenberg G, Martinez M, Hamberg M, Castresana C (2009). Diversity of the enzymatic activity in the lipoxygenase gene family of *Arabidopsis thaliana*. Lipids.

[26] Bannenberg G, Martínez M, Rodríguez MJ, López MA, Ponce De León I, Hamberg M (2009). Functional Analysis of α-DOX2, an Active α-Dioxygenase Critical for Normal Development in Tomato Plants. Plant Physiol.

[27] Wang Y, Oyaizu H (2009). Evaluation of the phytoremediation potential of four plant species for dibenzofuran-contaminated soil. J Hazard Mater.

[28] Zhang B, Zhang H, Jin J, Ni Y, Chen J (2012). PCDD/Fs-induced oxidative damage and antioxidant system responses in tobacco cell suspension cultures. Chemosphere.

[29] Jou JJ, Chung JC, Weng YM, Liaw SL, Wang MK (2007). Identification of dioxin and dioxin-like polychlorbiphenyls in plant tissues and contaminated soils. J Hazard Mater.

[30] Zhai G, Lehmler HJ, Schnoor JL (2010). Identification of hydroxylated metabolites of 3,3',4,4'-tetrachlorobiphenyl and metabolic pathway in whole poplar plants. Chemosphere.

[31] Li Y, Beisson F, Pollard M, Ohlrogge J (2006). Oil content of Arabidopsis seeds: the influence of seed anatomy, light and plant-to-plant variation. Phytochemistry.

[32] Costopoulou D, Vassiliadou I, Chrysafidis D, Bergele K, Tzavara E, Tzamtzis V, Leondiadis L (2010). Determination of PCDD/F, dioxin-like PCB and PAH levels in olive and olive oil samples from areas affected by the fires in summer 2007 in Greece. Chemosphere.

[33] Engwall M, Hjelm K (2000). Uptake of dioxin-like compounds from sewage sludge into various plant species assessment of levels using a sensitive bioassay. Chemosphere.

[34] Boucher J, Cengelli F, Trumbic D, Marison IW (2008). Sorption of hydrophobic organic compounds (HOC) in rapeseed oil bodies. Chemosphere.

[35] Boucher J, Steiner L, Marison IW (2007). Bio-sorption of atrazine in the press-cake from oilseeds. Water Res.

[36] Bolwell GP, Bozak K, Zimmerlin A (1994). Plant cytochrome P450. Phytochemistry.

[37] Taylor NL, Tan YF, Jacoby RP, Millar AH (2009). Abiotic environmental stress induced changes in the Arabidopsis thaliana chloroplast, mitochondria and peroxisome proteomes. J Proteomics.

[38] Coleman J, Blake-Kalff M, Davies E (1997). Detoxification of xenobiotics by plants: chemical modification and vacuolar compartmentation. Trends Plant Sci.

[39] Remy E, Duque P (2014). Beyond cellular detoxification: a plethora of physiological roles for MDR transporter homologs in plants. Front Physiol.

[40] Baxter I, Tchieu J, Sussman MR, Boutry M, Palmgren MG, Gribskov M (2003). Genomic comparison of P-type ATPase ion pumps in Arabidopsis and rice. Plant Physiol.

[41] Leier I, Jedlitschky G, Buchholz U, Cole SP, Deeley RG, Keppler D (1994). The MRP gene encodes an ATP-dependent export pump for leukotriene C4 and structurally related conjugates. J Biol Chem.

[42] Bellamy WT (1996). P-glycoproteins and multidrug resistance. Annual Rev Pharmacol Toxicol.

[43] Torija MJ, Beltran G, Novo M, Poblet M, Rozes N, Mas A (2003). Effect of organic acids and nitrogen source on alcoholic fermentation: study of their buffering capacity. J Agric Food Chem.

[44] Boyes DC, Zayed AM, Ascenzi R, McCaskill AJ, Hoffman NE, Davis KR (2001). Growth stage-based phenotypic analysis of Arabidopsis: a model for high throughput functional genomics in plants. Plant Cell.

[45] Yan Y, Shen L, Chen Y, Bao S, Thong Z, Yu H (2014). A MYB-domain protein EFM mediates flowering responses to environmental cues in Arabidopsis. Dev Cell.

[46] Seltmann MA, Stingl NE, Lautenschlaeger JK, Krischke M, Mueller MJ, Berger S (2010). Differential impact of lipoxygenase 2 and jasmonates on natural and stress-induced senescence in Arabidopsis. Plant Physiol.

[47] Keunen E, Remans T, Opdenakker K, Jozefczak M, Gielen H, Guisez Y (2013). A mutant of the Arabidopsis thaliana LIPOXYGENASE1 gene shows altered signalling and oxidative stress related responses after cadmium exposure. Plant Physiol Biochem.

[48] Bañuelos GR, Argumedo R, Patel K, Ng V, Zhou F, Vellanoweth RL (2008). The developmental transition to flowering in Arabidopsis is associated with an increase in leaf chloroplastic lipoxygenase activity. Plant Sci.

[49] Rodriguez Bañuelos C, Argumedo R, Patel K, Ng V, Zhou F, Vellanoweth RL (2009). The developmental transition to flowering in Arabidopsis is associated with an increase in leaf chloroplastic lipoxygenase activity. Plant Sci.

[50] Blee E, Boachon B, Burcklen M, Le Guedard M, Hanano A, Heintz D (2014). The reductase activity of the Arabidopsis caleosin RESPONSIVE TO DESSICATION20 mediates gibberellin-dependent flowering time, abscisic acid sensitivity, and tolerance to oxidative stress. Plant Physiol.

[51] Shen H, Han J, Tie X, Xu W, Ren Y, Ye C (2009). Polychlorinated dibenzo-p-dioxins/furans and polychlorinated biphenyls in human adipose tissue from Zhejiang Province. China Chemosphere.

[52] Katavic V, Reed DW, Taylor DC, Giblin EM, Barton DL, Zou J (1995). Alteration of seed fatty acid composition by an ethyl methanesulfonate-induced mutation in *Arabidopsis thaliana* affecting diacylglycerol acyltransferase activity. Plant Physiol.

[53] Murayama SY, Negishi Y, Umeyama T, Kaneko A, Oura T, Niimi M (2006). Construction and functional analysis of fatty acid desaturase gene disruptants in Candida albicans. Microbiology.

[54] Jiang ZY, Woollard AC, Wolff SP (1991). Lipid hydroperoxide measurement by oxidation of Fe2+ in the presence of xylenol orange. Comparison with the TBA assay and an iodometric method. Lipids.

[55] Gobel C, Feussner I, Hamberg M, Rosahl S (2002). Oxylipin profiling in pathogen-infected potato leaves. Biochim Biophys Acta.

[56] Hanano A, Burcklen M, Flenet M, Ivancich A, Louwagie M, Garin J (2006). Plant seed peroxygenase is an original heme-oxygenase with an EF-hand calcium binding motif. J Biol Chem.

[57] Czechowski T, Bari RP, Stitt M, Scheible WR, Udvardi MK (2004). Real-time RT-PCR profiling of over 1400 *Arabidopsis* transcription factors: unprecedented sensitivity reveals novel root- and shoot-specific genes. Plant J.

[58] Hu R, Fan C, Li H, Zhang Q, Fu YF (2009). Evaluation of putative reference genes for gene expression normalization in soybean by quantitative real-time RT-PCR. BMC Mol Biol.

