# Supplementary material for: Differential tissue accumulation of 2,3,7,8-Tetrachlorinated dibenzo-p-dioxin in Arabidopsis thaliana affects plant chronology, lipid metabolism and seed yield
Source: BMC Plant Biol. 2015 Aug 11;15:193. doi: 10.1186/s12870-015-0583-5 (PMC4531507; doi:10.1186/s12870-015-0583-5)
Supplement: Additional file 1: — Detection of TCDD in Arabidopsis tissues by HR/GC-MS. Diagrams indicate the presence of TCDD (Retention time ≈ 5.22) in the root (A, B and C) and in the shoot (D, E, and F) of 30-days old Arabidopsis exposed to various concentrations of TCDD 10, 50 and 100 ng L−1, respectively. G. TCDD standard. Three measurements were taken for three individual plants. Data are mean values ± SD (n = 6). (PDF 204 kb) [file 12870_2015_583_MOESM1_ESM.pdf]

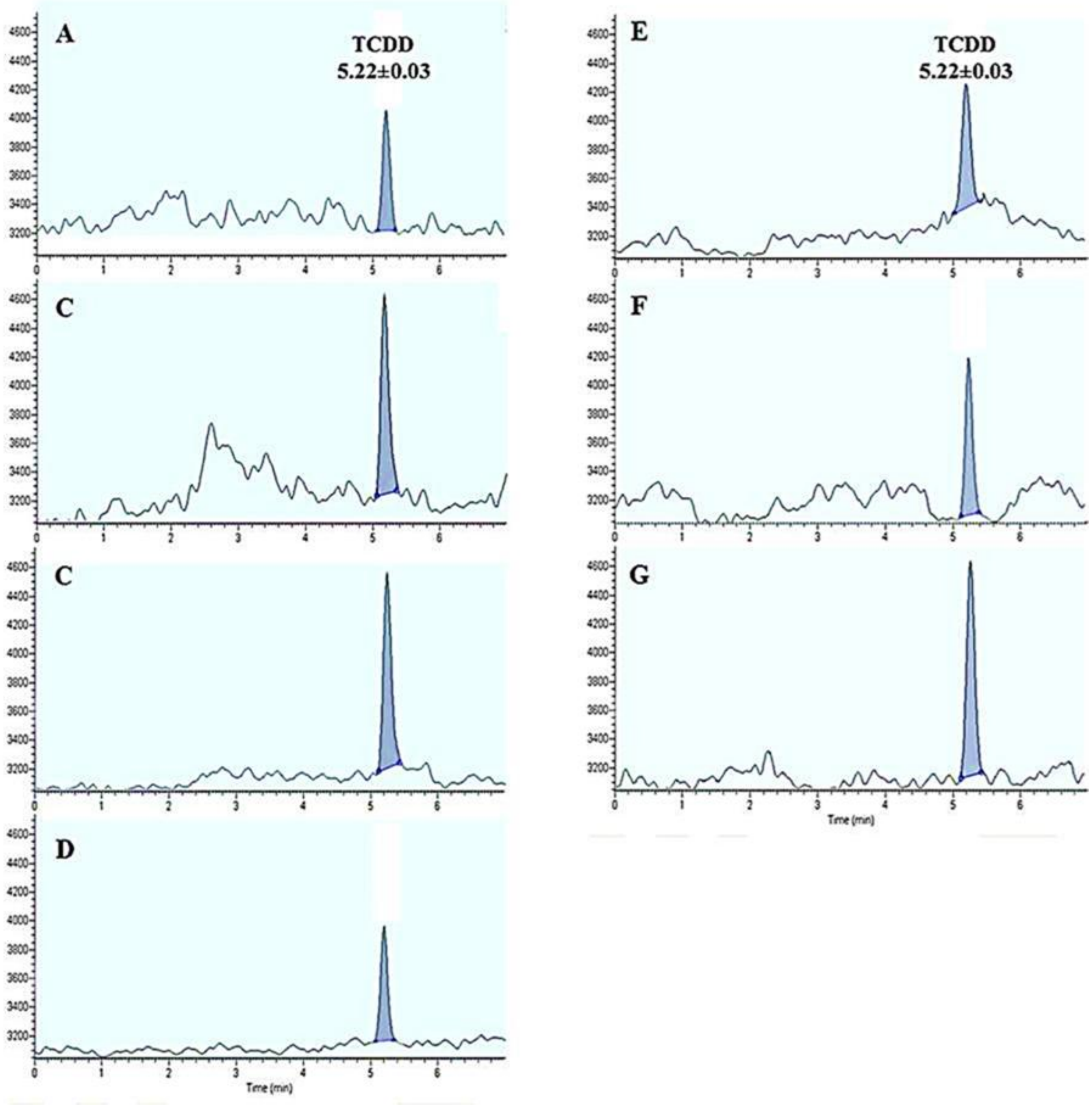

Additional file 1. Detection of TCDD in Arabidopsis tissues by HR/GC-MS. Diagrams indicate the presence of TCDD **(**Retention time ≈ 5.22**)** in the root (**A**, **B** and **C**) and in the shoot (**D**, **E**, and **F**) of 30-days old Arabidopsis exposed to various concentrations of TCDD 10, 50 and 100 ng L^{-1}, respectively. **G**. TCDD standard. Three measurements were taken for three individual plants. Data are mean values ± SD (*n* = 6).
